# Integrated Stress Responses to Bacterial Pathogenesis Patterns

**DOI:** 10.3389/fimmu.2018.01306

**Published:** 2018-06-07

**Authors:** Larissa O. C. P. Rodrigues, Rodrigo S. F. Graça, Leticia A. M. Carneiro

**Affiliations:** Laboratório de Inflamação e Imunidade, Instituto de Microbiologia Paulo de Góes, Universidade Federal do Rio de Janeiro, Rio de Janeiro, Brazil

**Keywords:** eukaryotic translation initiation factor 2 alpha, cellular stress, bacterial pathogens, heme-regulated eIF2α kinase, general control non-derepressible 2, PKR-like ER kinase, PKR

## Abstract

Activation of an appropriate innate immune response to bacterial infection is critical to limit microbial spread and generate cytokines and chemokines to instruct appropriate adaptive immune responses. Recognition of bacteria or bacterial products by pattern recognition molecules is crucial to initiate this response. However, it is increasingly clear that the context in which this recognition occurs can dictate the quality of the response and determine the outcome of an infection. The cross talk established between host and pathogen results in profound alterations on cellular homeostasis triggering specific cellular stress responses. In particular, the highly conserved integrated stress response (ISR) has been shown to shape the host response to bacterial pathogens by sensing cellular insults resulting from infection and modulating transcription of key genes, translation of new proteins and cell autonomous antimicrobial mechanisms such as autophagy. Here, we review the growing body of evidence demonstrating a role for the ISR as an integral part of the innate immune response to bacterial pathogens.

## Introduction

Microbial sensing by pattern recognition molecules (PRMs) triggers a robust innate immune response with the production of cytokines, chemokines, and antimicrobial factors (1–4). In the last decade, the concept that, in addition to microbial-associated molecular patterns recognition by PRMs, the host response can be tuned by the recognition of alterations in homeostasis induced by pathogens during progression of disease has been established (5–7). Such alterations on cell homeostasis allow the host to differentiate pathogenic organisms from those that do not represent a threat and, thus, adequate the immune responses to deal with the attack being mounted accordingly. It is interesting that despite the multitude of virulence mechanisms among bacterial species, most of them converge to few common “patterns of pathogenesis” that include membrane damage, access to the cytosol, disruption of cytoskeleton, and protein aggregation among others (5–10). In a sense, these patterns of pathogenesis would align with the concept of danger-associated molecular patterns (DAMPs), which are host molecules whose presence indicate that there has been tissue damage such as, for example, extracellular ATP or the chromatin-associated protein high-mobility group box 1 (11–13). But they are not exactly the same as DAMPs as these are molecules that are released as a result of cellular death and that are recognized by receptors on other cells whereas patterns of pathogenesis induce alterations of cell homeostasis during infection and provides the infected cell with information to mount a more refined response and to adapt and, in many cases recover from the insult.

In this context, the cellular mechanisms to sense and respond to stress can be regarded as an integral part of the innate immune response. The integrated stress response (ISR), a common adaptive pathway that eukaryotic cells activate in response to diverse stress stimuli is one such mechanism. The core event in this pathway is the phosphorylation of eukaryotic translation initiation factor 2 alpha (eIF2α) by one or more of four members of the eIF2α kinase family (6). The phosphorylation of eIF2α results in a marked decrease in global protein synthesis accompanied by the induction of selected genes, including the transcription factor ATF4, both of which are important to promote cellular recovery (6, 7, 14). This type of response to stress mediated by the eIF2α kinases, parallels those mediated by the mTOR pathway or by autophagy in the sense that are highly conserved signaling modules that regulate essential metabolic circuits, both in homeostatic and stress conditions, from yeast to mammals (6, 8, 15). In the context of an infection, the power of this type of “sensing system” relies on the fact that it does not recognizes pathogens *per se* but rather utilizes an ancient system that detects cellular stress/damage to sense insults that are caused by pathogenic bacteria regardless of its specific virulence factors.

In the present review, we focus on the emerging role of the ISR on host response to bacterial pathogens, which only recently began to be appreciated, in contrast to its well-established role in response to viruses. As obligate intracellular pathogens that highjack the host cell machinery to produce its own proteins, the link between viruses and the ISR is more obvious and more generally accepted. The impact of the ISR on viral infections has been extensively reviewed elsewhere (16–21). Here, we discuss recent data that implicate the ISR as an important component of cell autonomous anti-bacterial responses. As an emerging topic, there are still many gaps in our understanding of the mechanisms underlying this process but we believe that our current knowledge already provides a conceptual framework to work with. As much as we tried to bring together evidence of a role for ISR in different bacterial infections, this is by no means an exhaustive review.

## eIF2α and eIF2α-Kinases

Regulation of translation may be useful to coordinate several innate immune functions such as microbial sensing, microbial replication control, and induction of inflammatory cytokines. Translation shut down can help cells to cope with stress conditions and prevent further damage until the insult is gone. However, this happens in a context where cells still need to communicate that they are under attack in order to prevent infection spread and initiate adequate immune responses. Among metabolic sensors, eIF2α kinases have major roles in adjusting the protein synthesis machinery to enhance translation of mRNAs that are relevant to deal with the source of stress, including those induced by PRM activation, while shutting down the translation of unrelated proteins (6, 7, 21). This ability to screen and modulate host protein synthesis can affect the quality of the innate immune responses both at the transcriptional and translational levels. In addition, the gene expression program induced during ISR adjusts the stress response according to cellular context, nature, and intensity of stress stimuli (6, 7). Finally, although ISR is primarily a homeostatic-preserving program by which cells adapt to survive, severe and/or long-lasting stress can tip the balance toward cell death signaling by regulating the cell autonomous processes of autophagy and apoptosis (6, 7, 15).

The eIF2α kinases act as early responders to alterations in cellular homeostasis which is mainly due to the fact that these proteins are at the same time the sensors of stress and the kinases that phosphorylate eIF2α (6, 10, 20, 22). Each kinase dimerizes and autophosphorilates for full activation in response to distinct environmental and physiological types of stress. Double-stranded RNA (dsRNA)-dependent protein kinase (PKR) is activated mainly by dsRNA during viral infection but also by oxidative and ER stress, growth factor deprivation, cytokines, bacterial infections, and ribotoxic stress (23–27). Interestingly, caspase activity in the early stages of apoptosis was also shown to activate PKR, indicating a role for protein synthesis inhibition in apoptosis (28). PKR-like ER kinase (PERK) is activated by accumulation of unfolded proteins in the ER or perturbations in calcium homeostasis, cellular energy, or redox status (29–31). It has also been reported to respond to ATP depletion and subsequent sarcoplasmic/ER Ca^2+^-ATPase pump inhibition in the context of glucose deprivation in neuronal cells and in pancreatic β cells (32, 33). Heme-regulated eIF2α kinase (HRI) is a sensor for low levels of intracellular heme as well as arsenite-induced oxidative stress, heat shock, nitric oxide, 26S proteasome inhibition, and osmotic stress (34–37). This array of types of stress activate HRI independently of heme but require the presence of heat shock proteins HSP90 and HSP70 (37). General control non-derepressible 2 (GCN2) is highly conserved from yeasts to humans and is activated in response to amino acid deprivation when it binds to deacylated transfer RNAs (tRNAs) *via* histidyl-tRNA synthetase-related domain (38, 39). As one can appreciate, some types of stress can potentially activate more than one of these four kinases. Most likely, the eIF2α kinases act cooperatively to specifically tune cellular responses stress. Of note, all of these kinases have been reported to have roles independent of eIF2α phosphorylation but here we will focus on the ISR, which signals through eIF2α phosphorylation.

The common signaling hub for all the stress stimuli that activate ISR is phosphorylation of the subunit α of eIF2 on serine 51 (6, 10, 20, 22). eIF2 is constituted by three subunits (α, β, and γ). When bound to GTP and Met-tRNA_i_^Met^ (initiator methionyl-tRNA), eIF2 form a ternary complex that delivers the initiator tRNA to the 40S ribosomal subunit. eIF2 is released from the ribosome bound to a GDP and to be ready for another round of translation initiation, the eIF2 complex must be recycled back to its active GTP-bound form. The guanine nucleotide exchange factor eIF2B exchanges GDP for GTP on the γ subunit and maintains the levels of the ternary complex available for new rounds of translation. Under a variety of stress conditions, however, phosphorylation of the α subunit of eIF2 at Ser51 blocks general translation initiation, as it converts eIF2 to a competitive inhibitor of eIF2B by blocking the GDP–GTP exchange reaction and reducing the dissociation rate of eIF2 from eIF2B (6, 40, 41). Phosphorylation of eIF2α leads to a global arrest in translation but it does not affect all mRNA transcripts alike. A subset of mRNAs that contain upstream open reading frames and often encode proteins that are important for stress recovery and re-establishment of homeostasis have selective increased translation (6).

One of the genes that are upregulated following eIF2α phosphorylation is the transcription factor ATF4. Studies using ATF4-deficient mice have shown it has critical roles in the regulation of normal metabolic as well as redox processes such as regulation of obesity, glucose homeostasis, energy expenditure, and neural plasticity (42–44). Under stress conditions, increased ATF4 expression represents a signature of the ISR and is mainly due to translational control, as *Atf4* is one of those mRNAs that have its translation augmented upon eIF2α phosphorylation in contrast with the general translational arrest observed for most transcripts (6, 45). As a transcription factor, ATF4 can activate several transcriptional programs that will ultimately determine the cell fate—from cell death to re-establishment of homeostasis. The ability of ATF4 to interact with multiple other transcription factors allows it to generate distinct tailored responses to different types of cellular stress. Thus, despite ATF4 being a master common regulator of ISR, its target genes will be highly dependent on stress intensity and cellular context (45–47). For example, when acting in combination with ATF3, ATF4 is a part of a program that aims to re-establish cellular homeostasis and promote survival (48). Conversely, when interacting with CHOP, ATF4 promotes cell death following ER stress (49). In addition to the interacting partners that cooperate with ATF4 to promote transcription of target genes, another set of interacting partners prevent ATF4 transcriptional activity as is the case for PHD3 during hypoxia and TRIB3 during amino acid starvation and ER (50–52).

## ISR and Bacterial Infections

Eukaryotes have evolved in a context of constant interactions with prokaryotes and it is clear that the latter have contributed to shape those organisms throughout evolution. A human being harbors more than 1,000 bacterial species as part of their microbiota and interacts with another incalculable number of bacterial species during its lifetime (53). The vast majority of these interactions does not result in disease and, in many cases, they are actually beneficial to the hosts. However, despite representing less than 1% of the total number of estimated bacterial species in our planet, pathogenic bacteria still cause millions of deaths every year.

In general, those bacteria that are considered as pathogenic are the ones endowed with certain attributes that allow them to (1) colonize the host; (2) find a nutritionally compatible niche in the host body; (3) avoid, subvert, or circumvent the host innate and adaptive immune responses; (4) replicate, using host resources; and (5) exit and spread to a new host (54). However, even though some bacteria display very well-defined virulence attributes, the pathogenic potential of a given bacterium can only really be observed upon interaction with its host. The final outcome of an infection is never the result of bacterial virulence alone but rather a cross talk between the host and the pathogen. This complicates the definition of “true pathogen” as the same bacterial pathogen can have different impact in different individuals. Thus, for the host, it is critical to be able to assess the potential threat that a given pathogen represents in order to establish an appropriate response.

**Figure 1:**
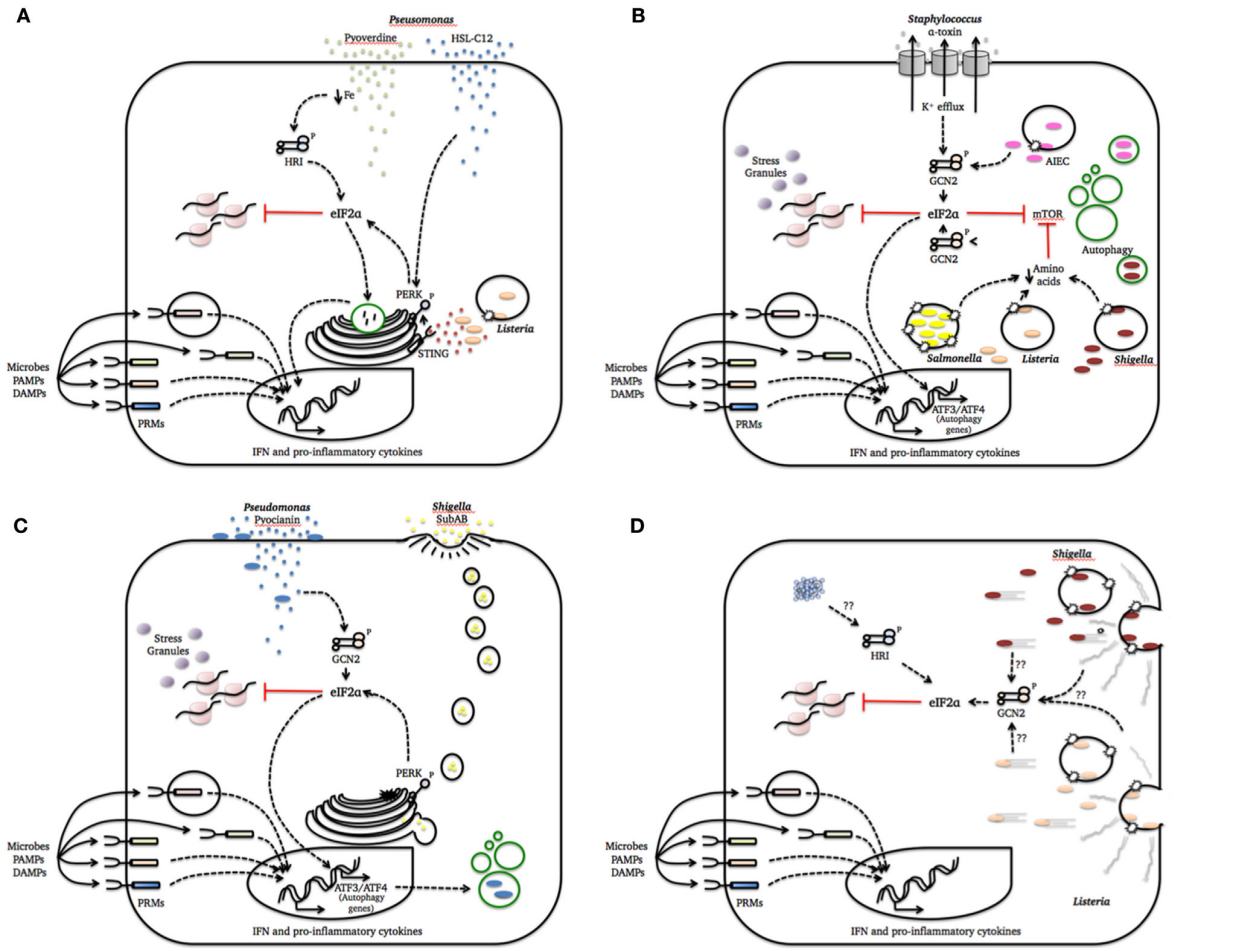
Integrated stress response (ISR) activation by bacterial pathogenesis patterns. This figure summarizes how cellular damage induced by different bacterial species is sensed by one or more eukaryotic translation initiation factor 2 alpha (eIF2α) kinases to activate defense mechanisms and homeostatic programs. We intentionally included a simplified representation of pattern recognition molecules (PRMs) recognition of microbes, PAMPs, and danger-associated molecular patterns (DAMPs) in all cartoons to strengthen the notion that these system act together to refine the cell response to the infection. **(A)** Pathogenesis pattern: bacterial growth; **(B)** pathogenesis pattern: membrane damage; **(C)** pathogenesis pattern: access to cytosol; **(D)** pathogenesis pattern: cytoskeleton disruption and protein aggregation.

During a bacterial infection, a multitude of signals exchanged by the two organisms establishes a cross talk that will ultimately determine the outcome of the infectious process. Many known bacterial virulence factors are only synthesized when bacteria go through major changes in metabolism in order to adapt to the dynamic conditions of the host environment (55). While doing that, bacterial pathogens may have profound effects on host cell homeostasis that, in turn, trigger cellular stress responses. Bellow, we will discuss how the ISR can be triggered by cellular alterations caused by bacterial infections and the impact of this response on host–pathogen interactions. The data discussed in the next sections are summarized in Figure 1.

## Bacterial Growth

The ability to survive and grow inside the host upon infection is one of the most common pathogenesis patterns as it represents the ability of a given pathogen to scape the host response and establish a replicative niche. For the host, being able to differentiate growing and dying bacteria, especially in the context of an acute infection, is key to mount a proper response. Molecules whose presence could indicate bacterial growth include peptidoglycan fragments released during bacterial cell division, quorum-sensing inducers that are produced once the bacterial population reaches a certain density and bacterial pyrophosphates such as HMB-PP (5, 56–58). Alternatively, instead of direct detection of a molecule, bacterial growth sensing could be achieved by sensing of altered local levels of cellular nutrients such amino acids or oxygen (59–61).

Recently, the definition of PAMPs has been updated to allow the classification of those produced specifically by living microorganisms, the so-called *Vita*-PAMPs, and those that represent the degradation products of dead microorganisms, named PAPMs-*postmortem* (PAMPs-PM), as two different categories that have different biological activities (10, 62). Moretti et al. (10) has recently identified cyclic-di-adenosine monophosphate (c-di-AMP), a second messenger that is produced by live Gram-positive bacteria, as a *Vita*-PAMP. The authors show that phagocytes are able to discriminate live and dead *Listeria innocua* by sensing this *Vita*-PAMP through the innate immune sensor stimulator of interferon genes resulting in ER stress, PERK and eIF2α phosphorylation. Subsequently, an autophagic response ensued to sequester stressed ER membranes and prevent stress-induced cell death while also inducing an IFN-dependent response. Importantly, this response was blunted in phagocytes lacking PERK. Finally, following *L. monocytogenes* infection, mice engineered to have PERK-deficient macrophages presented lower systemic levels of IFN-I and higher bacterial burden on both liver and spleen when compared with WT controls. In this model, at a cellular level, there was no difference between the response induced by live *L. innocua*, a non-pathogenic bacteria, and live *L. monocytogenes*, but there was differences when these were compared to dead bacteria. The response to any infection is multilayered and dependent on the interaction of multiple sensing systems—each one of these systems provides the cells with different information that when combined determine the cells response and, ultimately, its fate. In this case, the ISR provided the cells with the ability to distinguish live from dead bacteria, which is crucial to mount appropriate response even though it was not able to differentiate between a pathogenic from a non-pathogenic species. Of note, when the authors tested their hypothesis *in vivo*, they used only *L monocytogenes*, most likely because *L. innocua* would have been readily cleared given its lack of virulence and would have not generated any of the responses observed against *L. monocytogenes*. This, once again, illustrates how important the context is: in the natural course of a real infection, *L. innocua* would probably have never caused the systemic infection that *L. monocytogenes* does and would have not reached circulating phagocytic cells or the liver or the spleen. However, when given directly to these cells *in vitro*, it induced the same response that the bacterial species that would have encountered these cells during infection. It would also be interesting to investigate if this response is restricted to phagocytic cells or can occur in other cell types.

A contrasting study showed that PERK activation and IFN-I production by myeloid cells during infection with *L. monocytogens* or treatment with the pore-forming toxin LLO is actually detrimental to the host. In this model, the PERK pathway is amplified by IFN-I resulting in the activation of another eIF2α-kinase, PKR. This, in turn, served as an amplification loop for PERK-signaling leading to excessive ER stress and cell death. Consistent with this, mice deficient on CHOP, a pro-apoptotic factor that is downstream of PERK, are more resistant to *L. monocytogenes* infection than WT controls (63). This model could provide a partial explanation for why mice lacking IFN-I receptor have been consistently reported to be more resistant to *L. monocytogenes* than WT mice (64–66).

The opportunistic Gram-negative *Pseudomonas aeruginosa* causes both acute and chronic infections, especially in the respiratory tract (31, 67). Its ability to scape or subvert the host immune response constitutes its main virulence attribute. *P. aeruginosa* is able to form biofilms, a complex biological system that protects the bacteria from host immune defense mechanisms and promotes persistent infection. This bacterium coordinates the production of biofilms and other virulence factors using quorum sensing, a cell-to-cell communication system that allow bacteria to perceive their population density by producing and sensing diffusible signal molecules. One of the quorum-sensing auto inducers produced by *P. aeruginosa* to regulate gene expression and communicate is *N*-(30oxododecanoyl)-homoserin lactone (HSL-C12) (31, 67). HSL-C12 is a lipid-like diffusible molecule that has multiple effects on mammalian cells including apoptosis and release of Ca^2+^ from the ER stores. By perturbing ER homeostasis, HSL-C12 induces the activation of PERK and eIF2α phosphorylation resulting in protein synthesis inhibition (31). If in the one hand this inhibition results in increased NF-κB activation and transcription of pro-inflammatory genes because IκB re-synthesis is blocked, on the other hand it prevents the translation into proteins of the transcribed genes resulting in an overall downregulation of the host response and, thus, can be considered a pathogen scape mechanism. This would be one instance where the pathogen evolved to manipulate and take advantage of a cell host sensing system.

As mentioned above, nutrient availability is a critical limitation for invading microorganisms. Iron is a nutrient indispensable for growth of almost living organisms and is unlikely to be readily available for invading microorganisms resulting in fierce competition between host and pathogens (59). Like many other bacteria, *P. aeruginosa* has developed several mechanisms to acquire iron during infection. In a recent study, it was demonstrated that the iron-chelating siderophore pyoverdine produced by *P. aeruginosa* limits the concentration of iron in the cell medium resulting in the activation of HRI, eIF2α phosphorylation, and induction of *Gadd34* transcription in human bronchial epithelial cells. This response had cytoprotective effect and was turned off when the medium was supplemented with iron (61).

These few examples demonstrate that host cells can detect growth of bacteria by sensing molecules that accumulate as the number of bacteria increase including those that bacteria use to communicate with each other, such as quorum-sensing auto inducers and second messengers as well as molecules that bacteria use to acquire nutrients.

## Membrane Integrity

The detection of this type of stress is highly conserved. Damage of the plasma membrane is an archaic threat that needs to be faced with efficient cell autonomous defense mechanisms (5). Recently, a pivotal role for GCN2 in the response to membrane damage has been uncovered in different models. For example, it has been demonstrated that membrane permeabilization by the detergent digitonin induces a robust response characterized by GCN2 phosphorilation and ATF3 expression (68). In *Drosophila*, the damage caused by *Pseudomonas entomophila* in gut cells induces a starvation-like state, resulting in GCN2 and eIF2α phosphorylation and concomitant inhibition of the mTOR pathway by the AMP-activated kinase (AMPK). In this model, these two stress response pathways together shut down translation of new proteins and trigger innate immune responses (69).

In mammalian cells, disturbance of membrane integrity caused by bacterial pathogens can also trigger stress responses (8, 15, 68). Pore-forming toxins represent an important class of bacterial exoproducts that can induce membrane damage leading to stress responses (70). In human epithelial cells, the α-toxin produced by *Staphylococcus aureus* induces the formation of pores on cellular membranes resulting in potassium efflux, failure of nutrient transport and loss of ATP which, in turn, activates both GCN2 and the energy sensor AMPK, with subsequent eIF2α phosphorylation and mTORC1 deactivation (similar to what was reported in *Drosophila*) (15, 71). Low intracellular concentrations of potassium is known to trigger several responses in infected or stressed cells including the activation of inflammasomes and caspases (15, 71), as well as activation of multiple kinases such as p38 and CREB, in addition to the aforementioned AMPK and GCN2 (71–73). Activation of GCN2 induced by potassium efflux caused by membrane perforation indicates that cells may exploit the dependence of nutrient transport across the plasma membrane on physiological ion gradients to indirectly sense perturbations on ion concentration. Both removal of the pore from the plasma membrane by dynamin-dependent endocytosis and the metabolic reprogramming activated by the ISR are essential for cellular recovery as cells that are not able to activate this program are more susceptible to α-toxin (74, 75).

Invasive bacteria such as *Salmonella* Tiphymurium, *Shigella flexneri*, and *Listeria monocytogenes* also cause membrane damage during their internalization process. Similar to what was described above, all three bacteria trigger an acute intracellular amino acid starvation program that induces stress responses dependent on GCN2 and eIF2α phosphorylation at the same time as it disarms mTOR signaling unleashing an autophagic response (8, 68). However, the response that ensues is different for these three bacteria. (i) During infection with *S. flexneri*, a Gram-negative bacterium that escapes to and replicates in the host cell cytoplasm, amino acid starvation persists up to 4 h after infection allowing not only the induction of autophagy but also GCN2- and eIF2α-dependent formation of stress granules in the cytosol as well as reprogramming of the transcriptional response orchestrated by ATF3 (8, 76). (ii) *L. monocytogenes*, a Gram-positive bacterium that similar to *S. flexneri* escapes to and replicates in the cytosol, also triggers a state of amino acid starvation characterized by activation of GCN2, eIF2α phosphorylation, and transcriptional upregulation of ATF3. In this case, however, this response is very transient peaking at 1 h and is completely normalized after 4 h post-infection. The kinetics of this response parallels the kinetics of the pore-forming toxin LLO-dependent scape from the internalization vacuole and coincides with the maximal targeting of *L. monocytogenes* to autophagosomes (68). (iii) *Salmonella*, in contrast to the bacteria described above, remains in vesicles known as *Salmonella*-containing vacuoles (SCV) after its internalization. The damages to the SCV membranes trigger the same GCN2-dependent early amino acid starvation program described above. However, following *Salmonella* infection membrane integrity and cytosolic amino acid concentration are readily normalized allowing mTOR to be reactivated at the surface of the SCV and promoting bacterial scape from autophagy (8). Thus, these three model invasive bacteria all induce GCN2-dependent ISR during their entry processes but each one of them deal with it in different ways once again highlighting that bacteria have also evolved to counteract ISR-mediated responses.

Adherent-invasive *Escherichia coli* (AIEC), which is abnormally abundant in the intestinal mucosa of Crohn’s disease patients, also induces phosphorylation of GCN2 with subsequent eIF2α phosphorylation and increased ATF4 levels. Upon activation of this pathway, ATF4 binds to promoters of multiple autophagy-related genes including *MAP1LC3B, Becn1, SQSTM1, ATG3*, and *ATG7*. This is necessary to initiate autophagy and restrict bacterial growth as depleting cells from GCN2 resulted in impaired autophagy, increased bacterial replication, and elevated pro-inflammatory cytokine production both *in vitro* and *in vivo*. The authors go on to show that the GCN2–eIF2α–ATF4 pathway is activated in ileal biopsies from patients with noninflamed Crohn’s disease but not on those with inflamed Crohn’s disease, indicating that failure to activate this stress response could be one of the mechanisms contributing to active disease (77).

Thus, it appears that a nutrient sensor, GCN2, may also function as a sentinel of membrane integrity and that the responses it triggers are essential to prevent abyssal ATP loss and irreversible damage. In addition, in the case of invasive pathogens, this response might affect their ability to replicate within the host cell due to increase in autophagic activity as a consequence of amino acid starvation as well as production of inflammatory factors induced by the stress transcription factors ATF3 and ATF4.

## Access to Cytosol

Many pathogens are able to deliver molecules directly into the cytosol of host cell. This may be achieved by AB-toxins when the B subunit binds to specific receptors on the surface of the cells and translocates the active subunit A into the cell (78), by pore-forming toxins such as listeriolysin O (mentioned above) and streptolysin O (70), or secretion systems such as the type III secretion systems of *Yersinia* and *Salmonella* (79), the type IV secretion system of *Legionella, Coxiella*, and *Brucella* (80), and the type VI secretion system of *Pseudomonas* and *Vibrio* (81, 82).

Shiga-toxigenic *E. coli* produces Shiga toxin (Stx) 1 and 2 that cause hemorrhagic colitis and hemolytic uremic syndrome. A newly described toxin, namely subtilase cytotoxin (SubAB), was shown to bind to and be internalized by target cells through clathrin-, lipid rafts-, and actin-dependent pathways. Once it reaches the ER, SubAB cleaves the chaperone Bip/Grp78 initiating an ER-stress induced ISR resulting in cytotoxicity. This response also included the formation of stress granules induced not only by PERK but also as a result of PKR activation (14).

Yang et al. (83) show that *P. aeruginosa* infection induces a strong activation of the GCN2–eIF2α–ATF4 pathway that is largely dependent on production of pyocianin during initial infection and that ultimately results in bacterial clearance through autophagy. Pyocianin is a cell permeable toxin considered to be a major virulence factor for *P aeruginosa*. *In vivo*, in rats, infection with a mutant bacterial strain that does not produce pyocianin and, thus, does not activate of the GCN2–eIF2α–ATF4 pathway results in higher number of colony-forming units in the lungs, more extensive alveolar wall thickening and higher mortality when compared to infection with the WT strain. Although indirect, these data suggest a role for the ISR in preventing prolonged infection and immunopathology. Interestingly, reduction of pyocianin production by *P. aeruginosa* in chronic airways infections has been associated with better host adaptation and worse outcomes in cystic fibrosis patients (84).

## Actin Cytoskeleton Disruption

Another common feature employed by various highly divergent pathogenic bacterial species is the disruption of the host cell cytoskeleton. Invasive bacteria such as *S. flexneri, L. monocytogenes, Mycobacterium marinum*, and Rickettsial species exploit the actin-based motility to move inside the cell and from one cell to the other without never being exposed to immune defenses outside the cells (5, 85). Other bacterial pathogens, such as *E. coli* and *Citrobacter freundii*, produce hallmark attaching and effacing lesions that are characterized by localized destruction of the brush border villi of enterocytes, intimate attachment of bacteria to the residual apical membrane and formation of a dense plaque of actin cytoskeletal filaments beneath adherent bacteria that is essential for their pathogenesis (86, 87). Finally, some pathogens manipulate host actin cytoskeleton to either induce their own uptake or to avoid phagocytosis (88–90).

Polysomes, mRNAs, elongations factors, and aminoacyl-tRNA synthetases are found associated with actin filaments indicating that the cytoskeleton might actually act as a platform to facilitate the assembly of components involved translation (91–93). GCN2 has been recently implicated as a sensor of F-actin depolymerization. Disruption of the actin cytoskeleton by drugs such as latruculin-B and cytochalasin-D induces GCN2 activation followed by eIF2α phosphorylation, attenuation of global translation, and augmented ATF4 and CHOP expression (94). In nutrient-replete cells, GCN2 is kept in a latent state by the interaction with other proteins such as the eukaryotic elongation factor 1A (eEF1A) that delivers aminoacyl-tRNAs to ribosomes during the elongation step of protein synthesis (95, 96). During starvation periods, however, uncharged tRNA displaces eEF1A from GCN2 allowing its autophosphorilation and eIF2α phosphorylation (97, 98). Another binding partner of eEF1A is F-actin. In yeasts, the same mutations that affect binding of eEF1A to aminoacyl-tRNAs also result in actin binding and buding defects that lead to GCN2-dependent eIF2α phosphorylation (99, 100). Thus, it has been proposed that upon F-actin disruption eEF1A is displaced from GCN2 and bound to F-actin leaving GCN2 free to initiate the ISR (94). In addition, F-actin disruption also leads to deacylated tRNA accumulation, which in turn might also contribute to the activation of GCN2 resulting in global protein synthesis arrest and reduction of amino acylated tRNA levels (94).

As mentioned above, two invasive pathogens—*L. monocytogenes* and *S. flexneri*—that exploit the actin cytoskeleton of the cell to move around the cell and infect neighboring cells were shown to induce a GCN2-dependent starvation program as a consequence of membrane damage. It is possible that disruption of F-actin could impede the proper function of amino acid transporters on the plasma membrane triggering this response. In summary, infection with *L. monocytogenes* and *S. flexneri* could potentially activate GCN2 in multiple ways: when bacteria escape from the vacuole into the cytosol causing membrane damage (as it has been experimentally demonstrated) or by disrupting the actin cytoskeleton.

## Protein Aggregation

Heme-regulated eIF2α kinase is able to sense and respond to a variety of types of cellular stress including heme deprivation, oxidative stress, heat shock, and proteasome inhibition, all of which are known to result in accumulation of misfolded protein aggregates in the cytosol (35, 36, 101, 102). As it has been previously shown that infection with bacterial pathogens trigger the formation of large PRM oligomeric complexes in the cytosol, one may speculate that this is the common feature among all these types of stresses that is actually sensed by HRI (103–106). This could serve as a sensing system to monitor misfolding of large protein complexes and formation of toxic aggregates in the cytosol and trigger damage control mechanisms such as ISR and autophagy.

## Concluding Remarks

Even at a single cell level, the response to an infection is multilayered and involves sensing, effector, and homeostatic mechanisms. Each one of these elements has, in itself, multiple layers of complexity and, together, they generate a full-blown response. Sensing of microbes or their products by PRMs is pivotal and activates robust inflammatory responses. Since the discovery of PRMs, there has been much debate on how the cells can tailor the response to specific pathogens using a limited number of receptors that recognize structures that are present in many different microorganisms, including non-pathogenic. This can be achieved by different means including the combinatorial effect of several PRMs (107), the compartmentalization of PRMs that only allows recognition of certain PAMPs when presented in specific compartments of the cell (108) and the sensing of *vita*-PAMPs versus PAMPs-PM (62). The recognition of pathogenesis patterns by the ISR represents another layer in the host response. Sensing alterations on homeostasis and cell damaged caused by infection can instruct the host to generate a more refined and specific response while triggering protective gene expression programs that enable cells to recover from the initial stress and re-establish homeostasis. Given its origins early on evolution, stress responses may actually represent an ancient innate defense mechanism against invading pathogens.

In this review, we discussed evidence showing that the ISR can have an important role in shaping the autonomous cell response to bacteria with varying levels of virulence. In this context, the ISR acts in concert with other sensing systems to adequate the response to the threat. Thus, the ISR during bacterial infection cannot be analyzed isolated from the context. This generates a complexity that represents a challenge for dissecting the precise role and the relevance of each component in the final response. While there are still many gaps to be filled before we have a more comprehensive overview, the picture that emerges is that the ISR can influence the quality of the response initiated by innate immune recognition.

For the most part, the studies discussed in this review show that several bacteria are able to activate or manipulate the ISR during infection, through different eIF2α kinases and signaling pathways, resulting in specific transcriptional programs. However, in many cases, it is yet to be defined how this affects the outcome of the infection. In some cases, it is clear that it can affect the ensuing immune response. For example, ISR activation in phagocytic cells infected by *Listeria* was shown to be critical for IFN-I production and bacterial clearance. On the other hand, eIF2α phosphorylation induced by the HSL-C12 from *P. aeruginosa* results in downregulation of translation of pro-inflammatory cytokines such as IL-6 and KC. We believe that understanding how the ISR can affect qualitatively the response to a given pathogen is a major avenue for future work. In this sense, as we progress, it would be important to determine how the ISR impact on how cells communicate infection to the neighboring cells as well as to immune cells and how this can qualitatively affect the immune response, including in subsequent exposures to the same pathogen. Finally, it would be interesting to see if the homeostatic adaptations during infection can lead to persistent alterations in the infected cell rendering it more resistant to following infections.

The interplay between ISR and autophagy is also a common theme in most of the studies mentioned here. Interestingly, two studies discussed above showed that in the absence of the GCN2–eIF2α–ATF4-autophagy pathway, opportunistic bacteria such as AIEC and *P. aeruginosa* establish persistent infection that perpetuate inflammation contributing to worsen the pathology in Crohn’s disease and cystic fibrosis, respectively. It will interesting to investigate how ISR could affect the development of chronic complex diseases that are known to have a microbial component to it such as the two mentioned above.

As we mentioned above, co-evolution of pathogens and their hosts have shaped (and continue to do so) their interactions. In this constant arms race, both sides try to adapt in order to survive. Thus, it should come as no surprise that some bacteria might be able to scape or even take advantage of the ISR to manipulate the host cell response. Indeed, being able to subvert host responses is part of the very definition of what a pathogen is. In this case, failure to activate the ISR properly could lead the host to underestimate the infectious threat. As it is well documented for several viruses, we expect that as our knowledge increases, we will uncover many bacterial strategies to tamper with the ISR.

Because of the significant overlap between the eIF2α kinases in addition to the complexity of many host–pathogen interactions, at this point, it is difficult to clear define the role of each of the ISR sensors in response to bacterial pathogens and most likely a combination of them are responsible for an appropriate response. Future work will help us understand how these pathways are activated and manipulated by bacterial pathogens and how can we use this knowledge to develop new treatments to prevent or cure infection. For example, if we are able to safely increase the signals generated by cells of the innate immunity by manipulating the ISR, we might be able to improve the adaptive immunity generated by vaccines.

## Author Contributions

LR, RG, and LC wrote the manuscript. LC edited and revised the final version of the manuscript.

## Conflict of Interest Statement

The authors declare that the research was conducted in the absence of any commercial or financial relationships that could be construed as a potential conflict of interest.

## References

[1] Medzhitov R. Recognition of microorganisms and activation of the immune response. Nature (2007) 449:819–26. 10.1038/nature06246 17943118

[2] Carneiro LA Magalhaes JG Tattoli I Philpott DJ Travassos LH Nod-like proteins in inflammation and disease. J Pathol (2008) 214:136–48. 10.1002/path.2271 18161746

[3] Iwasaki A Medzhitov R. Control of adaptive immunity by the innate immune system. Nat Immunol (2015) 16:343–53. 10.1038/ni.3123 25789684 PMC4507498

[4] Kumar S Ingle H Prasad DV Kumar H. Recognition of bacterial infection by innate immune sensors. Crit Rev Microbiol (2013) 39:229–46. 10.3109/1040841X.2012.706249 22866947

[5] Vance RE Isberg RR Portnoy DA. Patterns of pathogenesis: discrimination of pathogenic and nonpathogenic microbes by the innate immune system. Cell Host Microbe (2009) 6:10–21. 10.1016/j.chom.2009.06.007 19616762 PMC2777727

[6] Pakos-Zebruca K Koryga I Mnich K Ljujic M Samali A Gorman AM The integrated stress response. EMBO Rep (2016) 17:1374–95. 10.15252/embr.201642195 27629041 PMC5048378

[7] Argüello RJ Rodrigues CR Gatti E Pierre P. Protein synthesis regulation, a pillar of strength for innate immunity? Curr Opin Immunol (2015) 32:28–25. 10.1016/j.coi.2014.12.001 25553394

[8] Tattoli I Sorbara MT Vuckovic D Ling A Soares F Carneiro LA Amino acid starvation induced by invasive bacterial pathogens triggers an innate host defense program. Cell Host Microbe (2012) 11:563–75. 10.1016/j.chom.2012.04.012 22704617

[9] Keestra-Gounder AM Byndloss MX Seyffert N Young BM Chávez-Arroyo A Tsai AY NOD1 and NOD2 signaling links ER stress with inflammation. Nature (2016) 532:394–7. 10.1038/nature17631 27007849 PMC4869892

[10] Moretti J Roy S Bozec D Martinez J Chapman JR Ueberheide B STING senses microbial viability to orchestrate stress-mediated autophagy of the endoplasmic reticulum. Cell (2017) 171:809–823.e13. 10.1016/j.cell.2017.09.034 29056340 PMC5811766

[11] Andersson U Yang H Harris H. High-mobility group box 1 protein (HMGB1) operates as an alarmin outside as well as inside cells. Semin Immunol (2018) 5323:30076–83. 10.1016/j.smim.2018.02.011 29530410

[12] Fleshner M Crane CR. Exosomes, DAMPs and miRNA: features of stress physiology and immune homeostasis. Trends Immunol (2017) 38:768–76. 10.1016/j.it.2017.08.002 28838855 PMC5624844

[13] Adinolfi E Giuliani AL De Marchi E Pegoraro A Orioli E Di Virgilio F The P2X7 receptor: a main player in inflammation. Biochem Pharmacol (2018) 151:234–44. 10.1016/j.bcp.2017.12.021 29288626

[14] Tsutsuki H Yahiro K Ogura K Ichimura K Iyoda S Ohnishi M Subtilase cytotoxin produced by locus of enterocyte effacement-negative Shiga-toxigenic Escherichia coli induces stress granule formation. Cell Microbiol (2016) 18:1024–40. 10.1111/cmi12565 26749168 PMC10068837

[15] von Hoven G Kloft N Neukirch C Ebinger S Bobkiewicz W Weis S Modulation of translation and induction of autophagy by bacterial exoproducts. Med Microbiol Immunol (2012) 201:401–18. 10.1007/s00430-012-0271-0 PMC3470817 22991039

[16] Thomas MG Loschi M Desbats MA Boccaccio GL. RNA granules: the good, the bad and the ugly. Cell Signal (2011) 23:324–34. 10.1016/j.cellsig.2010.08.011 20813183 PMC3001194

[17] Jheng JR Ho JY Horng JT. ER stress, autophagy, and RNA viruses. Front Microbiol (2014) 5:388–95. 10.3389/fmicb.2014.00388 25140166 PMC4122171

[18] Montero H Trujillo-Alonso V. Stress granules in the viral replication cycle. Viruses (2011) 3:2328–3238. 10.3390/v3112328 22163347 PMC3230854

[19] Carrasco L Sanz MA González-Almela E. The regulation of translation in alphavirus-infected cells. Viruses (2018) 10:70. 10.3390/v10020070 29419763 PMC5850377

[20] McCormick C Khaperskyy DA. Translation inhibition and stress granules in the antiviral immune response. Nat Rev Immunol (2017) 17:647–60. 10.1038/nri.2017.63 28669985

[21] Hoang HD Graber TE Alain T. Battling for ribosomes: translational control at the forefront of the antiviral response. J Mol Biol (2018) 2836:30362–70. 10.1016/j.jmb.2018.04.040 29746850

[22] Donnelly N Gorman AM Gupta S Samali A. The eIF2α kinases: their structures and functions. Cell Mol Life Sci (2013) 70:3493–511. 10.1007/s00018-012-1252-6 23354059 PMC11113696

[23] Clemens MJ Elia A. The double-stranded RNA-dependent protein kinase PKR: structure and function. J Interferon Cytokine Res (1997) 17:503–24. 10.1089/jir.1997.17.503 9335428

[24] Lemaire PA Anderson E Lary J Cole JL. Mechanism of PKR activation by dsRNA. J Mol Biol (2008) 381:351–60. 10.1016/j.jmb.2008.05.056 18599071 PMC2570377

[25] Shimazawa M Hara H. Inhibitor of double stranded RNA-dependent protein kinase protects against cell damage induced by ER stress. Neurosci Lett (2006) 409:192–5. 10.1016/j.neulet.2006.09.074 17055645

[26] Garcia MA Gil J Ventoso I Guerra S Domingo E Rivas C Impact of protein kinase PKR in cell biology: from antiviral to antiproliferative action. Microbiol Mol Biol Rev (2006) 70:1032–60. 10.1128/MMBR.00027-06 17158706 PMC1698511

[27] Zhou HR He K Landgraf J Pan X Pestka JJ Direct activation of ribosome-associated double-stranded RNA-dependent protein kinase (PKR) by deoxynivalenol, anisomycin and ricin: a new model for ribotoxic stress response induction. Toxins (2014) 6:3406–25. 10.3390/toxins6123406 25521494 PMC4280541

[28] Saelens X Kalai M Vandenabeele P Translation inhibition in apoptosis: caspase-dependent PKR activation and eIF2-alpha phosphorylation. J Biol Chem (2001) 276:41620–8. 10.1074/jbc.M103674200 11555640

[29] Korennykh A Walter P. Structural basis of the unfolded protein response. Annu Rev Cell Dev Biol (2012) 28:251–77. 10.1146/annurev-cellbio-101011-155826 23057742

[30] Wang M Kaufman RJ. Protein misfolding in the endoplasmic reticulum as a conduit to human disease. Nature (2016) 529:326–35. 10.1038/nature17041 26791723

[31] Grabiner MA Fu Z Wu T Barry KC Scharzer C Machen TE *Pseudomonas aeruginosa* quorum-sensing molecule homoserine lactone modulates inflammatory signaling through PERK and eI-F2. J Immunol (2014) 193:1459–67. 10.4049/jimmunol.1303437 24990083 PMC4113911

[32] de la Cadena SG Hernandez-Fonseca K Camacho-Arroyo I Massieu L. Glucose deprivation induces reticulum stress by the PERK pathway and caspase-7- and calpain-mediated caspase-12 activation. Apoptosis (2014) 19:414–27. 10.1007/s10495-013-0930-7 24185830

[33] Moore CE Omikorede O Gomez E Willars GB Herbert TP PERK activation at low glucose concentration is mediated by SERCA pump inhibition and confers preemptive cytoprotection to pancreatic beta-cells. Mol Endocrinol (2011) 25:315–26. 10.1210/me.2010-0309 21193559 PMC3070211

[34] Ill-Raga G Tajes M Busquets-Garcia A Ramos-Fernandez E Vargas LM Bosch-Morato M Physiological control of nitric oxide in neuronal BACE1 translation by heme-regulated eIF2alpha kinase HRI induces synaptogenesis. Antioxid Redox Signal (2015) 22:1295–307. 10.1089/ars.2014.6080 25706765

[35] McEwen E Kedersha N Song B Scheuner D Gilks N Han A Heme-regulated inhibitor kinase-mediated phosphorylation of eukaryotic translation initiation factor 2 inhibits translation, induces stress granule formation, and mediates survival upon arsenite exposure. J Biol Chem (2005) 280:16925–33. 10.1074/jbc.M412882200 15684421

[36] Yerlikaya A Kimball SR Stanley BA. Phosphorylation of eIF2alpha in response to 26S proteasome inhibition is mediated by the haem-regulated inhibitor (HRI) kinase. Biochem J (2008) 412:579–88. 10.1042/BJ20080324 18290760 PMC2842126

[37] Lu L Han AP Chen JJ. Translation initiation control by heme-regulated eukaryotic initiation factor 2alpha kinase in erythroid cells under cytoplasmic stresses. Mol Cell Biol (2001) 21:7971–80. 10.1128/MCB.21.23.7971-7980.2001 11689689 PMC99965

[38] Castilho BA Shanmugam R Silva RC Ramesh R Himme BM Sattlegger E. Keeping the eIF2 alpha kinase Gcn2 in check. Biochim Biophys Acta (2014) 1843:1948–68. 10.1016/j.bbamcr.2014.04.006 24732012

[39] Vazquez de Aldana CR Wek RC Segundo PS Truesdell AG Hinnebusch AG. Multicopy tRNA genes functionally suppress mutations in yeast eIF-2 alpha kinase GCN2: evidence for separate pathways coupling GCN4 expression to unchanged tRNA. Mol Cell Biol (1994) 14:7920–7793. 10.1128/MCB.14.12.7920 7969132 PMC359331

[40] Kepp O Semeraro M Bravo-San Pedro JM Bloy N Buqué A Huang X eIF2α phosphorylation as a biomarker of immunogenic cell death. Semin Cancer Biol (2015) 33:86–92. 10.1016/j.semcancer.2015.02.004 25749194

[41] Kashiwagi K Ito T Yokoyama S. Crystal structure of eIF2B and insights into eIF2-eIF2B interactions. FEBS J (2017) 284:868–74. 10.1111/febs.13896 27627185

[42] Wortel IMN van der Meer LT Kilberg MS van Leeuwen FN. Surviving stress: modulation of ATF4-mediated stress responses in normal and malignant cells. Trends Endocrinol Metab (2017) 28:794–806. 10.1016/j.tem.2017.07.003 28797581 PMC5951684

[43] Wei N Zhu LQ Liu D. ATF4: a novel potential therapeutic target for Alzheimer’s disease. Mol Neurobiol (2015) 52:1765–70. 10.1007/s12035-014-8970-8 25381575

[44] Wang C Guo F. Effects of activating transcription factor 4 deficiency on carbohydrate and lipid metabolism in mammals. IUBMB Life (2012) 64:226–30. 10.1002/iub.605 22223547

[45] B’Chir W Maurin AC Carraro V Averous J Jousse C Muranishi Y The eIF2alpha/ATF4 pathway is essential for stress-induced autophagy gene expression. Nucleic Acids Res (2013) 41:7683–99. 10.1093/nar/gkt563 23804767 PMC3763548

[46] Harding HP Zhang Y Zeng H Novoa I Lu PD Calfon M An integrated stress response regulates amino acid metabolism and resistance to oxidative stress. Mol Cell (2003) 11:619–33. 10.1016/S1097-2765(03)00105-9 12667446

[47] Karpinski BA Morle GD Huggenvik J Uhler MD Leiden JM. Molecular cloning of human CREB-2: an ATF/CREB transcription factor that can negatively regulate transcription from the cAMP response element. Proc Natl Acad Sci U S A (1992) 89:4820–4. 10.1073/pnas.89.11.4820 1534408 PMC49179

[48] Ohoka N Yoshii S Hattori T Onozaki K Hayashi H. TRB3, a novel ER stress-inducible gene, is induced via ATF4-CHOP pathway and is involved in cell death. EMBO J (2005) 24:1243–55. 10.1038/sj.emboj.7600596 15775988 PMC556400

[49] Wang Q Mora-Jensen H Weniger MA Perez-Galan P Wolford C Hai T ERAD inhibitors integrate ER stress with an epigenetic mechanism to activate BH3-only protein NOXA in cancer cells. Proc Natl Acad Sci U S A (2009) 106:2200–5. 10.1073/pnas.0807611106 19164757 PMC2629785

[50] Hiwatashi Y Kanno K Takasaki C Goryo K Sato T Torii S PHD1 interacts with ATF4 and negatively regulates its transcriptional activity without prolyl hydroxylation. Exp Cell Res (2011) 317:2789–99. 10.1016/j.yexcr.2011.09.005 21951999

[51] Koditz J Nesper J Wottawa M Stiehl DP Camenisch G Franke C Oxygen-dependent ATF-4 stability is mediated by the PHD3 oxygen sensor. Blood (2007) 110:3610–7. 10.1182/blood-2007-06-094441 17684156

[52] Jousse C Deval C Maurin AC Parry L Cherasse Y Chaveroux C TRB3 inhibits the transcriptional activation of stress-regulated genes by a negative feedback on the ATF4 pathway. J Biol Chem (2007) 282:15851–61. 10.1074/jbc.M611723200 17369260

[53] Nava GM Stappenbeck TS. Diversity of the autochthonous colonic microbiota. Gut Microbes (2011) 2:99–104. 10.4161/gmic.2.2.15416 21694499 PMC3225773

[54] Finlay BB Falkow S. Common themes in microbial pathogenicity. Microbiol Rev (1989) 53:210–30. 2569162 10.1128/mr.53.2.210-230.1989 PMC372728

[55] Orji FA Ugbogu OC Ugbogu EA Barbabosa-Pliego A Monroy JC Elghandour MMMY Pathogenic flora composition and overview of the trends used for bacterial pathogenicity identifications. Microb Pathog (2018) 5:139–46. 10.1016/j.micpath.2018.05.006 29738815

[56] Carneiro LA Travassos LH Soares F Tattoli I Magalhaes JG Bozza MT *Shigella* induces mitochondrial dysfunction and cell death in nonmyleoid cells. Cell Host Microbe (2009) 5:123–36. 10.1016/j.chom.2008.12.011 19218084

[57] Zimmermann S Wagner C Müller W Brenner-Weiss G Hug F Prior B Induction of neutrophil chemotaxis by the quorum-sensing molecule N-(3-oxododecanoyl)-L-homoserine lactone. Infect Immun (2006) 74:5687–92. 10.1128/IAI.01940-05 16988244 PMC1594900

[58] Hintz M Reichenberg A Altincicek B Bahr U Gschwind R Kollas AK Identification of (E)-4-hydroxy-3-methyl-but-2-enyl pyrophosphate as a major activator for human γδ T cells in Escherichia coli. FEBS Letters (2001) 509:317–22. 10.1016/S0014-5793(01)03191-X 11741609

[59] Ganz T Nemeth E. Iron homeostasis in host defence and inflammation. Nat Rev Immunol (2015) 15:500–10. 10.1038/nri3863 26160612 PMC4801113

[60] Vasil ML Ochsner UA. The response of *Pseudomonas aeruginosa* to iron: genetics, biochemistry and virulence. Mol Microbiol (1999) 34:399–341. 10.1046/j.1365-2958.1999.01586.x 10564483

[61] van ‘t Wout EF van Schadewijk A van Boxtel R Dalton LE Clarke HJ Tommassen J Virulence factors of *Pseudomonas aeruginosa* induce both the unfolded protein and integrated stress responses in airway epithelial cells. PLoS Pathog (2015) 11(6):e1004946. 10.1371/journal.ppat.1004946 26083346 PMC4471080

[62] Blander JM Sander LE. Beyond pattern recognition: five immune checkpoints for scaling the microbial threat. Nat Rev Immunol (2012) 12:215–25. 10.1038/nri3167 22362354

[63] Valderrama C Clark A Urano F Unanue ER Carrero JA Listeria monocytogenes induces an interferon-enhanced activation of the integrated stress response that is detrimental for resolution of infection in mice. Eur J Immunol (2017) 47:830–40. 10.1002/eji.201646856 28267207 PMC5450196

[64] Carrero J Calderon B Unanue E. Type I interferon sensitizes lymphocytes to apoptosis and reduces resistance to *Listeria* infection. J Exp Med (2004) 200:535–40. 10.1084/jem.20040769 15302900 PMC2211931

[65] O’Connell RM Saha SK Vaidya SA Bruhn KW Miranda GA Zarnegar B Type I interferon production enhances susceptibility to *Listeria monocytogenes* infection. J Exp Med (2004) 200:437–45. 10.1084/jem.20040712 15302901 PMC2211937

[66] Auerbuch V Brockstedt DG Meyer-Morse N O’Riordan M Portnoy DA. Mice lacking the type I interferon receptor are resistant to *Listeria monocytogenes*. J Exp Med (2004) 200:527–33. 10.1084/jem.20040976 15302899 PMC2211930

[67] Rasamiravaka T El Jaziri M. Quorum-sensing mechanisms and bacterial response to antibiotics in *P. aeruginosa*. Curr Microbiol (2016) 73:747–53. 10.1007/s00284-016-1101-1 27449213

[68] Tattoli I Sorbara MT Yang C Tooze SA Philpott DJ Girardin SE. *Listeria* phospholipases subvert host autophagic defenses by stalling pre-autophagosomal structures. EMBO J (2013) 32:3066–78. 10.1038/emboj.2013.234 24162724 PMC3844955

[69] Chakrabarti S Liehl P Buchon N Lemaitre B. Infection-induced host translational blockage inhibits immune responses and epithelial renewal in the *Drosophila* gut. Cell Host Microbe (2012) 12:60–70. 10.1016/j.chom.2012.06.001 22817988

[70] Dal Peraro M van der Goot FG. Pore-forming toxins: ancient, but never really out of fashion. Nat Rev Microbiol (2016) 14:77–92. 10.1038/nrmicro.2015.3 26639780

[71] Kloft N Neukirch C Bobkiewicz W Veerachato G Busch T von Hoven G Pro-autophagic signal induction by bacterial pore-forming toxins. Med Microbiol Immunol (2010) 199:299–309. 10.1007/s00430-010-0163-0 20454906 PMC2955911

[72] Kloft N Busch T Neukirch C Weis S Boukhallouk F Bobkiewicz W Pore-forming toxins activate MAPK p38 by causing loss of cellular potassium. Biochem Biophys Res Commun (2009) 385:503–6. 10.1016/j.bbrc.2009.05.121 19497299

[73] Gonzalez MR Bischofberger M Freche B Ho S Parton RG van der Goot FG. Pore-forming toxins induce multiple cellular responses promoting survival. Cell Microbiol (2011) 13:1026–43. 10.1111/j.1462-5822.2011.01600.x 21518219

[74] Kloft N Neukirch C Von Hoven G Bobkiewicz W Weis S Boller K A subunit of eukaryotic translation initiation factor 2alpha-phosphatase (CreP/PPP1R15B) regulates membrane traffic. J Biol Chem (2012) 287:35299–317. 10.1074/jbc.M112.379883 22915583 PMC3471756

[75] von Hoven G Neukirch C Meyenburg M Füser S Petricna MB Rivas AJ eIF2α confers cellular tolerance to S. aureus α0-toxin. Front Immunol (2015) 6:383.103389/fimmu.2015.00383 26284068 10.3389/fimmu.2015.00383 PMC4515601

[76] Travassos LH Carneiro LA Ramjeet M Hussey S Kim YG Magalhães JG Nod1 and Nod2 direct autophagy by recruiting ATG16L1 to the plasma membrane at the site of bacterial entry. Nat Immunol (2010) 11:55–62. 10.1038/ni.1823 19898471

[77] Bretin A Carriere J Dalmasso G Bergougnoux A B’chir W Maurin AC Activation of the EIF2AK4-EIF2A/eIF2a-ATF4 pathway triggers autophagy response to Crohn disease-associated adherent-invasive *Escherichia coli* infection. Autophagy (2016) 12:770–83. 10.1080/15548627.2016.1156823 26986695 PMC4854551

[78] do Vale A Cabanes D Sousa S. Bacterial toxins as pathogen weapons against phagocytes. Front Microbiol (2016) 7:42–9. 10.3389/fmicb.2016.00042 26870008 PMC4734073

[79] Deng W Marshall NC Rowland JL McCoy JM Worrall LJ Santos AS Assembly, structure, function and regulation of type III secretion systems. Nat Rev Microbiol (2017) 15:323–37. 10.1038/nrmicro.2017.20 28392566

[80] Grohmann E Christie PJ Waksman G Backert S. Type IV secretion in Gram-negative and Gram-positive bacteria. Mol Microbiol (2018) 107:455–71. 10.1111/mmi.13896 29235173 PMC5796862

[81] Sana TG Berni B Bleves S. The T6SSs of *Pseudomonas aeruginosa* strain PAO1 and their effectors: beyond bacterial-cell targeting. Front Cell Infect Microbiol (2016) 6:61–6. 10.3389/fcimb.2016.00061 27376031 PMC4899435

[82] Joshi A Kostiuk B Rogers A Teschler J Pukatzki S Yildiz FH. Rules of engagement: the type VI secretion system in *Vibrio cholerae*. Trends Microbiol (2017) 25:267–79. 10.1016/j.tim.2016.12.003 28027803 PMC5365375

[83] Yang ZS Ma LQ Zhu K Yan JY Bian L Zhang KQ Pseudomonas toxin pyocyanin triggers autophagy: implications for pathoadaptive mutations. Autophagy (2016) 12:1015–28. 10.1080/15548627.2016.1170256 27159636 PMC4922443

[84] Rada B Leto TL. Pyocyanin effects on respiratory epithelium: relevance in *Pseudomonas aeruginosa* airway infections. Trends Microbiol (2013) 21:73–81. 10.1016/j.tim.2012.10.004 23140890 PMC3565070

[85] Gouin E Welch MD Cossart P. Actin-based motility of intracellular pathogens. Curr Opin Microbiol (2005) 8:35–45. 10.1016/j.mib.2004.12.013 15694855

[86] Gaytán MO Martínez-Santos VI Soto E González-Pedrajo B. Type three secretion system in attaching and effacing pathogens. Front Cell Infect Microbiol (2016) 6:129–35. 10.3389/fcimb.2016.00129 27818950 PMC5073101

[87] Schmidt MA. LEEways: tales of EPEC, ATEC and EHEC. Cell Microbiol (2010) 12:1544–52. 10.1111/j.1462-5822.2010.01518.x 20716205

[88] Galan JE Wolf-Watz H. Protein delivery into eukaryotic cells by type III secretion machines. Nature (2006) 444:567–73. 10.1038/nature05272 17136086

[89] Viboud GI Bliska JB. *Yersinia* outer proteins: role in modulation of host cell signaling responses and pathogenesis. Annu Rev Microbiol (2005) 59:69–89. 10.1146/annurev.micro.59.030804.121320 15847602

[90] Heasman SJ Ridley AJ. Mammalian Rho GTPases: new insights into their functions from in vivo studies. Nat Rev Mol Cell Biol (2008) 9:690–701. 10.1038/nrm2476 18719708

[91] Kim S Coulombe PA. Emerging role for the cytoskeleton as an organizer and regulator of translation. Nat Rev Mol Cell Biol (2010) 11:75–81. 10.1038/nrm2818 20027187

[92] Sattlegger E Chernova TA Gogoi NM Pillai IV Chernoff YO Munn AL. Yeast studies reveal moonlighting functions of the ancient actin cytoskeleton. IUBMB Life (2014) 66:538–45. 10.1002/iub.1294 25138357 PMC4176509

[93] Sotelo-Silveira J Crispino M Puppo A Sotelo JR Koenig E. Myelinated axons contain beta-actin mRNA and ZBP-1 in periaxoplasmic ribosomal plaques and depend on cyclic AMP and F-actin integrity for in vitro translation. J Neurochem (2008) 104:545–57. 10.1111/j.1471-4159.2007.04999.x 17961153

[94] Silva RC Sattlegger E Castilho BA. Perturbations in actin dynamics reconfigure protein complexes that modulate GCN2 activity and promote an eIF2 response. J Cell Sci (2016) 129:4521–33. 10.1242/jcs.194738 27852836

[95] Garriz A Qiu H Dey M Seo E-J Dever TE Hinnebusch AG. A network of hydrophobic residues impeding helix alphaC rotation maintains latency of kinase Gcn2, which phosphorylates the alpha subunit of translation initiation factor 2. Mol Cell Biol (2009) 29:1592–607. 10.1128/MCB.01446-08 19114556 PMC2648240

[96] Lageix S Zhang J Rothenburg S Hinnebusch AG. Interaction between the tRNA-binding and C-terminal domains of Yeast Gcn2 regulates kinase activity in vivo. PLoS Genet (2015) 11:e1004991. 10.1371/journal.pgen.1004991 25695491 PMC4335047

[97] Marton MJ Crouch D Hinnebusch AG. GCN1, a translational activator of GCN4 in *Saccharomyces cerevisiae*, is required for phosphorylation of eukaryotic translation initiation factor 2 by protein kinase GCN2. Mol Cell Biol (1993) 13:3541–56. 10.1128/MCB.13.6.3541 8497269 PMC359824

[98] Sattlegger E Hinnebusch AG. Separate domains in GCN1 for binding protein kinase GCN2 and ribosomes are required for GCN2 activation in amino acid-starved cells. EMBO J (2000) 19:6622–33. 10.1093/emboj/19.23.6622 11101534 PMC305848

[99] Gross SR Kinzy TG. Improper organization of the actin cytoskeleton affects protein synthesis at initiation. Mol Cell Biol (2007) 27:1974–89. 10.1128/MCB.00832-06 17178834 PMC1820457

[100] Perez WB Kinzy TG Translation elongation factor 1A mutants with altered actin bundling activity show reduced aminoacyl-tRNA binding and alter initiation via eIF2alpha phosphorylation. J Biol Chem (2014) 289:20928–38. 10.1074/jbc.M114.570077 24936063 PMC4110299

[101] Han AP Yu C Lu L Fujiwara Y Browne C Chin G Heme-regulated eIF2alpha kinase (HRI) is required for translational regulation and survival of erythroid precursors in iron deficiency. EMBO J (2001) 20:6909–18. 10.1093/emboj/20.23.6909 11726526 PMC125753

[102] Ranu RS Regulation of protein synthesis in rabbit reticulocyte lysates: the hemeregulated protein kinase (HRI) and double stranded RNA induced protein kinase (dRI) phosphorylate the same site(s) on initiation factor eIF-2. Biochem Biophys Res Commun (1979) 91:1437–44. 10.1016/0006-291X(79)91227-0 526314

[103] Caruso R Warner N Inohara N Nunez G. NOD1 and NOD2: signaling, host defense, and inflammatory disease. Immunity (2014) 41:898–908. 10.1016/j.immuni.2014.12.010 25526305 PMC4272446

[104] Gaudet RG Sintsova A Buckwalter CM Leung N Cochrane A Li J Cytosolic detection of the bacterial metabolite HBP activates TIFA-dependent innate immunity. Science (2015) 348:1251–5. 10.1126/science.aaa4921 26068852

[105] Hou F Sun L Zheng H Skaug B Jiang QX Chen ZJ. MAVS forms functional prion-like aggregates to activate and propagate antiviral innate immune response. Cell (2011) 146:448–61. 10.1016/j.cell.2011.06.041 21782231 PMC3179916

[106] Maekawa S Ohto U Shibata T Miyake K Shimizu T. Crystal structure of NOD2 and its implications in human disease. Nat Commun (2016) 7:11813–8. 10.1038/ncomms11813 27283905 PMC4906405

[107] Creagh EM O’Neill LA. TLRs, NLRs and RLRs: a trinity of pathogen sensors that co-operate in innate immunity. Trends Immunol (2006) 27:352–7. 10.1016/j.it.2006.06.003 16807108

[108] Brubaker SW Bonham KS Zanoni I Kagan JC. Innate immune pattern recognition: a cell biological perspective. Annu Rev Immunol (2015) 33:257–90. 10.1146/annurev-immunol-032414-112240 25581309 PMC5146691

